# Visual cues enhance effectiveness of pheromone-baited traps for the corn earworm moth, *Helicoverpa zea* (Lepidoptera: Noctuidae)

**DOI:** 10.1093/jee/toaf024

**Published:** 2025-02-13

**Authors:** Charles A Kwadha, Ahmed M Saveer, Ayako Wada-Katsumata, Dominic D Reisig, Gabriel P Hughes, Ring T Cardé, Coby Schal

**Affiliations:** Department of Entomology and Plant Pathology, North Carolina State University, Raleigh, NC, USA; Department of Entomology and Plant Pathology, North Carolina State University, Raleigh, NC, USA; Department of Entomology and Plant Pathology, North Carolina State University, Raleigh, NC, USA; Department of Entomology and Plant Pathology, North Carolina State University, Raleigh, NC, USA; Department of Entomology, University of California Riverside, Riverside, CA, USA; Department of Entomology, University of California Riverside, Riverside, CA, USA; Department of Entomology and Plant Pathology, North Carolina State University, Raleigh, NC, USA

**Keywords:** bollworm, night vision, pheromones, moths, pests

## Abstract

Mate-finding and host localization by nocturnal moths are predominantly mediated by olfactory signals and cues, respectively. Nevertheless, some nocturnal moth species rely on olfactory and visual cues to locate resources, such as flowers. Although traps are indispensable for the detection and monitoring of corn earworm moths, *Helicoverpa zea* (Boddie), a generalist and highly destructive crop pest, the role of visual cues in locating a pheromone source is poorly understood. Here, we investigated whether trap color influences the trap catch of the corn earworm. We showed that trap design affected male *H. zea* trap catch, with Hartstack-type traps being more effective than bucket traps, and more *H. zea* males were trapped in light-colored traps (white, yellow). However, under the dim ambient night conditions, when *H. zea* males fly, it is unlikely that they can discern trap colors. Instead, it is probable that *H. zea* males discriminate traps on the basis of their gray-scale reflectance, ranging from white to black. We found a positive correlation between trap captures and the relative luminance of dyed cheesecloth fabrics that we wrapped around Hartstack traps. Our findings suggest that at night, *H. zea* integrates the visual contrast between the trap and foliage background (ie apparency of the trap) in locating sex pheromone-baited traps.

## Graphical Abstract

**Graphical Abstract:**
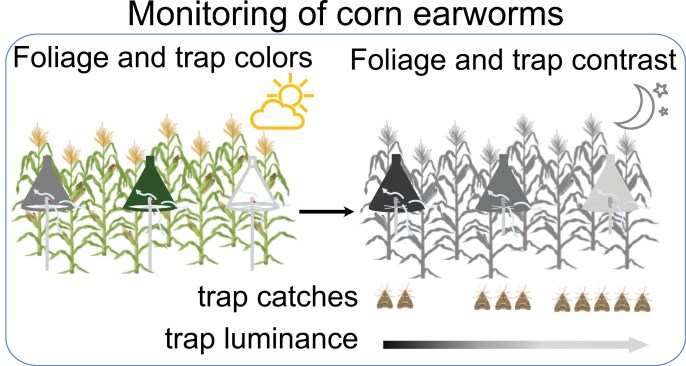

## Introduction

The corn earworm (CEW), *Helicoverpa zea* (Boddie) (Lepidoptera: Noctuidae), widely present in the tropical and temperate regions of the Americas, is a common and destructive pest, especially of corn (Cartwright 1939, Zimmerman and Fletcher 1955, Hardwick 1965), but also of soybean, cotton, and sorghum (Reay-Jones 2019). Given the damage associated with infestations of major crops by CEW and its long-range migration (Hartstack et al. 1983, Goodenough et al. 1988), early detection is important to inform integrated pest management decisions. Pheromone-baited traps are widely used to detect and monitor invasive pest population densities such as heliothine moths that are major agricultural pests (Hartstack et al. 1979). For CEW, traps are baited with the female-produced sex pheromone composed of (*Z*)-11-hexadecenal (Z11-16:Ald) and (*Z*)-9-hexadecenal (Z9-16:Ald) as the major and secondary components, respectively (Klun et al. 1980, Sparks et al. 1980, Pope et al. 1984). Additionally, (*Z*)-7-hexadecenal and hexadecanal are produced by females, but their addition to the 2-component mixture does not increase male responses and trap catch (Klun et al. 1980, Sparks et al. 1980, Vetter and Baker 1984).

**Fig. 1.**
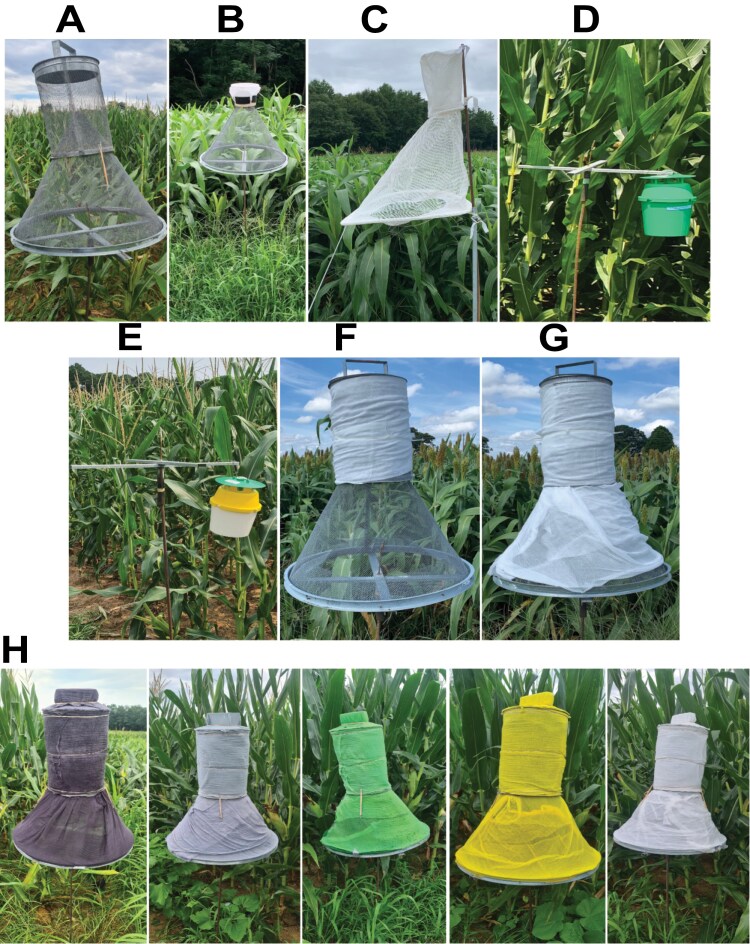
Trap design and color variation used in field trapping experiments with *Helicoverpa zea*. The various traps used in this study, including (A) Hartstack-wire trap, (B) Hartstack-wire-white plastic trap, (C) Scentry Heliothis trap, (D) Universal unicolor bucket trap, and (E) Universal multicolor bucket trap. (F–H) Improvised Hartstack traps wrapped in dyed cheesecloth fabric. In (H) from left to right: Black, Gray, Green, Yellow, White.

About 5 decades ago, high demand for more efficient lures and traps for monitoring CEW stimulated studies on pheromone composition, component ratios, dispensers, and trap design, as well as combinations of these variables. However, substantial variation and disparities were recorded across studies (Klun et al. 1980, Sparks et al. 1980, 1981, Vetter and Baker 1984, Gauthier et al. 1991). In separate studies and disregarding other aspects, the Hartstack trap (also known as Texas cone trap), the wind-vane trap, and the electrocutor grid trap were found to be the most effective at trapping males (Mitchell et al. 1978, Hartstack et al. 1979, Sparks et al. 1980, 1981). However, due to financial and practical constraints associated with trap use, the Hartstack trap, Heliothis net trap, and the bucket trap (also known as the Universal moth trap or Unitrap, hereafter Universal bucket trap) have been favored for monitoring heliothine moths including CEW (Lopez Jr et al. 1994, Guerrero et al. 2014, Olmstead and Shelton 2016, Djaman et al. 2019). The Hartstack trap has a cone made of rigid metallic gray wire mesh (Fig. 1A). A collapsible version of this trap made of white nylon, the Heliothis or Scentry net trap, was later developed (Fig. 1C). The Universal bucket trap often comes as a unicolor trap (forest green) or multicolor trap (green cover, yellow top and white bottom) (Fig. 1D,E, respectively) (Hartstack et al. 1979, Laurent and Frérot 2007).

Moth trapping relies heavily on olfactory inputs, since most pest moth species are nocturnal. To our knowledge, no studies have systematically investigated the effect of trap color on trapping of heliothine species, but such studies have been done with other moth species. In the beet armyworm (*Spodoptera exigua* (Hübner)), the fall armyworm (*Spodoptera frugiperda* (J. E. Smith)), the velvetbean caterpillar (*Anticarsia gemmatalis* Hübner) and the Mexican rice borer (*Eoreuma loftini* (Dyar)), more moths were trapped in multicolored bucket traps and Scentry Heliothis traps than Universal unicolor bucket traps or Hartstack traps (Mitchell et al. 1976, 1989, Shaver et al. 1991, Lopez Jr 1998, Saveer et al. 2023). More tobacco budworm moths, *Chloridea* (formerly *Heliothis*) *virescens* (F.), were caught in either green or orange painted carton traps than in yellow or white-painted carton traps (Hendricks et al. 1972). Since CEW has a spectral sensitivity pattern similar to *S. frugiperda* and *A. gemmatalis*, which are most sensitive at wavelengths of 530 and 520 nm, respectively (Agee 1973, Mitchell et al. 1989), and CEW prefers to oviposit on cotton leaves (Braswell et al. 2019), which have a light reflectance at 525 to 575 nm (Taft et al. 1969), we hypothesized that trap color and relative luminance might influence the trap catch of pheromone-baited traps. With on-going efforts to improve the efficiency of traps for early detection, assessing population dynamics and distribution of CEW, and direct pest management (mass trapping, attract-and-kill), it is imperative to investigate the effects of trap color on moth captures.

## Materials and Methods

### Traps

We hypothesized that trap color and trap design influence the attraction of CEW to pheromone-baited traps. We compared the following traps in field trapping experiments (Fig. 1):

a. Hartstack wire trap. This trap is standard in CEW monitoring programs (Hartstack et al. 1979, Gauthier et al. 1991). In previous work with heliothine moths, we used a white plastic top (Groot et al. 2018). Therefore, 4 versions of this trap were tested: an all-wire trap (Hartstack-wire) (Fig. 1A), a wire trap with a white plastic top (Hartstack-wire-white plastic) (Fig. 1B), a wire bottom and with the top wrapped in dyed white cheesecloth (Hartstack-wire-white cheesecloth) (Fig. 1F), and an all-wire trap wrapped in dyed cheesecloth (Hartstack-white-white cheesecloth) (Fig. 1G). In a preliminary experiment, we spray-painted Hartstack traps with white enamel paint (Rust-Oleum Flat White 7590838, Vernon Hills, IL, USA). However, odors associated with the spray paint appeared to interfere with trapping. Therefore, we dyed white cheesecloth (DeRoyal Grade 40 Medium, Burlap Fabric, Chicago, IL, USA) using White, Truly Green, Lemon Yellow, Pearl Gray, and Black following the manufacturer's directions (Rit Dye Liquid, Nakoma Products, Bridgeview, IL, USA). The stained fabrics were fixed using Rit ColorStay Dye Fixative, left to dry for 2 d, laundered without detergent, and air dried. These fabrics were used to wrap Hartstack traps (Fig. 1H), as described below.
b. Heliothis trap (Fig. 1C). This is a nylon version of the Hartstack trap, consisting of a collapsible two-chambered net (Guerrero et al. 2014) (hereafter Scentry Heliothis trap), that is also commonly used to monitor heliothine moths.
c. Universal unicolor bucket trap (all components forest green color) (GL/IP-2351-03; Green; Great Lakes IPM, Vestaburg, MI, USA) (Fig. 1D). Preliminary observations in southern California (GPH and RTC) indicated that more CEW were captured in these traps than in either clear Universal bucket traps or Hartstack traps.
d. Universal multicolor bucket trap (green cover, yellow top, and white bottom) (GL/IP-2352; Yellow/White; Great Lakes IPM, Vestaburg, MI, USA) (Fig. 1E). This trap was more effective than the Hartstack trap at trapping *S. frugiperda* (Saveer et al. 2023).

### Field Experiments

We carried out field experiments in the summers of 2022 and 2023 in corn fields located along Inwood Road (35° 43′ 45.6024″N, 78° 40′ 54.0762″ W) and sorghum fields along Mid Pines Road (35° 44′ 4.416″N, 78° 42′ 35.7588″ W) at the Lake Wheeler Field Research Station, Raleigh, North Carolina, USA. Early in our field season, corn and sorghum plants were at the tasseling and booting stages, respectively. The plants developed through the maturity and soft dough stages. For all the fields, linear experimental blocks were set at the edges of crop fields.

In 2022, we carried out 2 experiments. During the first experiment, conducted between 17 and 23 August, the lunar phases were from waning gibbous (between a full moon and a half-moon) and waning crescent (between the last quarter and a new moon). The average daily temperature was 23.1 ± 0.4 °C (SEM) and the relative humidity (RH) was 82.9 ± 2.3% (Supplementary Fig. S1A). Three trap designs were tested: the Universal bucket trap, Hartstack trap (Saveer et al. 2023), and Scentry Heliothis trap. Two color types of the universal bucket trap were tested: unicolor and multicolor bucket traps. Hartstack traps were of 2 designs: Hartstack-wire and Hartstack-wire-white plastic. Five treatments (trap types) consisting of the unicolor bucket, multicolor bucket, Hartstack-wire, Hartstack-wire-white plastic, and Scentry Heliothis traps were set within each of 2 linear blocks and rotated nightly so each trap type was in each of the 5 trap positions within a block for one night (*n* = 10 to 13 per trap type). Except for the Scentry Heliothis trap, we connected cross bars to rebars driven into the ground to hold the traps in position. The bucket and Hartstack traps were mounted on the cross bars. Within a block, traps were set out in a line and spaced 20 m apart. Similarly, the blocks were also in a line with 20 m between blocks. Traps were baited with commercial Pherocon CEW lures (red septa) (Trécé Inc., Adair, OK, USA) containing Z11-16:Ald and Z9-16:Ald. Septa were held in position using lure baskets in Universal bucket traps or alligator clips mounted on the cross bars for the Hartstack traps. For the Scentry Heliothis traps, the clips were attached to the string across the opening of the bottom cone. An insecticidal strip (Hercon Vaportape II containing 10% 2,2-dichlorovinyl dimethyl phosphate (DDVP), Hercon Environmental Co., Emigsville, PA, USA) was added to each bucket trap to kill trapped moths. The base of each trap was set at approximately 1.5 m above the ground and about 0.25 m below the corn canopy. Traps were serviced daily by collecting trapped insects, recording the number of CEW moths, and rotating the traps and associated lures as a unit to the next trap position within the block to minimize positional effects.

In the second 2022 trapping experiment, between 3 and 14 September, the moon phases were from the first quarter through the full moon to waning gibbous, and the average daily temperature was 24.1 ± 0.6 °C and the RH was 80.4 ± 3.6% (Supplementary Fig. S1A). We tested 5 treatments consisting of the multicolor bucket, Hartstack-wire, Scentry Heliothis, a modified Hartstack-wire-white cheesecloth trap, and Hartstack-white-white cheesecloth trap. The unicolor bucket trap was abandoned based on the results of the first trapping experiment. The Hartstack-wire-white-plastic was also discarded to minimize potential reflectance noise from the plastic. The traps were set in a linear block and rotated nightly for 12 nights so each trap type was in each of the 5 trap positions within a block for one night (*n* = 12 per trap type).

Three trials were conducted in 2023. The first trial was performed between 25 and 28 July, during the first quarter and waxing gibbous (toward full moon) phases, at an average daily temperature of 26.8 ± 3.1 °C and RH of 76.5 ± 1.7% (Supplementary Fig. S1B). We compared the trapping efficacy of Universal unicolor and multicolor bucket traps (Fig. 1) in a paired experimental design using 2 traps per block and 3 blocks. The trap pair within each block was rotated between the 2 positions nightly for 4 d (*n* = 12 per trap type). The second experiment was conducted 1–6 August, starting at full moon and progressing through waning gibbous. The average daily temperature was 24.2 ± 0.6 °C and the RH was 73.5 ± 2.5% (Supplementary Fig. S1B). We compared the Hartstack-wire and 5 Hartstack traps wrapped in different dyed cheesecloths including gray, black, white, yellow, and green (hereafter, Hartstack-gray-gray, Hartstack-black-black, Hartstack-white-white, Hartstack-yellow-yellow and Hartstack-green-green, respectively, denoting the colors of the base cone and the top wire trap). Both the bottom cone and top trap were wrapped in the same color cheesecloth (Fig. 1H). We deployed the 6 trap types in 2 blocks with nightly rotation of traps for 6 nights within each block (*n* = 12 per trap type). During the third experiment conducted 9–14 August, the moon phase started at last quarter and progressed through waning crescent, the average daily temperature was 26.4 ± 0.5 °C and the RH was 75.5 ± 2.0% (Fig. S1B). We repeated the second experiment without the Hartstack-gray-gray and Hartstack-black-black traps in a sorghum field off Mid Pines Road. The 4 trap types within each of 2 linear blocks were spaced, serviced, and rotated daily for 6 d (*n* = 12 per trap type), as described above.

### Cheesecloth Luminance Analysis

Spectral reflectance of a surface is invariant (remains constant under day and night light conditions) (Hurlbert 2007, Warrant and Somanathan 2022), so it offers a proxy to establish the relative luminance (ie brightness of a surface). To determine the relative luminance of the cheesecloth fabrics, we scanned strips of the gray, black, green, yellow and white fabrics (15.25 × 2.5 cm) used in the field trapping. Scanning was done in grayscale mode (Multifunction printer-Bizhub C360i, Konica Minolta Inc., Mexico). Using ImageJ (Schneider et al. 2012), the mean gray value of each fabric section (2.5 × 1.25 cm) (Supplementary Table S1, Supplementary Fig. S2) was determined from an 8-bit size of the scanned image. The values were then used to calculate the relative luminance, *Y,* using the equation Y= 0.2126∗R + 0.7152∗G + 0.0722∗B; where *R*, *G*, and *B* are the reflectance values of the surface under red, green, and blue channels, respectively.

### Data Analysis

The average daily weather conditions, recorded by the North Carolina State Climate Office (https://products.climate.ncsu.edu/cardinal/request, accessed October 10, 2023), were plotted in Microsoft 365 Office Excel. To compare trap catches between crops and possible interaction between crop and position of traps in summer 2022, we used linear mixed model (*lmm*) followed by analysis of anova (*anova*) from the model. Trap catches of male CEW per trap were expressed as percentages relative to the total number of male CEW trapped per block. Because the data were not normally distributed, we arcsine-transformed the data before analysis. Linear mixed model (*lmm*) in R package “*lmerTest*” (Kuznetsova et al. 2017), fitted with restricted maximum likelihood was used to analyze data from unicolor *vs.* multicolor bucket traps and the 6-colored Hartstack trap experiment. A Tukey test with Bonferroni adjustment was applied for the post-hoc multiple comparison. A similar approach with the 4-colored Hartstack trap experiment yielded a singularity problem (predictor variables had an exact linear relationship between them—perfect multicollinearity). Instead, we used a generalized linear mixed model (*glmm*) followed by estimated mean marginal means (*emmeans*) through R package “*emmean*s” (Lenth et al. 2018) with Tukey adjustment for pairwise comparison. For both the *lmm* and *glmm*, trap type and position were treated as fixed and random variables respectively. To establish whether there was a correlation between the trap captures and the relative luminance, data from the second and third experiments (2023) exclusive of Hartstack-wire trap catches, were used to calculate Pearson’s correlation. All data were analyzed in R programming language by the R Core Team (2021) at *α* = 0.05. We used the R package “*Tidyverse*” (Wickham 2017) and Affinity Designer for visualization.

## Results

### CEW Catches Vary with Trap Type and Design

**Fig. 2.**
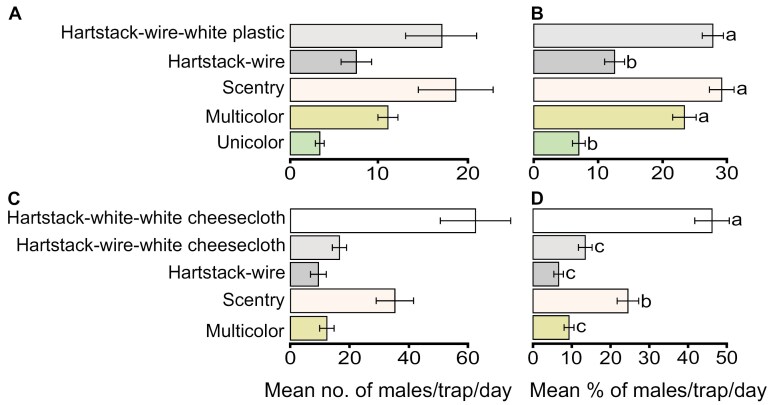
Effect of trap design and color on trap catch of *Helicoverpa zea* males in summer 2022. All traps were baited with sex pheromone. The bar plots show (A) the mean number and (B) the mean percentage of males caught per trap per day on 17–23 August in sorghum and corn fields (*n* = 25), (C) the mean number and (D) mean percentage of males caught per trap per day in a sorghum field on 3–14 September (*n* = 12 per trap type). Different letters indicate significant differences following *lmm* and post-hoc analysis with *α=* 0.05. Measures of variation represent ±SEM.

During the first field trapping experiment in 2022, a total of 1,437 CEW males were caught between 17 and 23 August, at relatively low moon light. The captures per trap type were not significantly different between the 2 crops, sorghum and corn (Table 1). Similarly, the interaction between trap type and the position of the traps within each block did not have a significant effect (Table 1). Therefore, we pooled the CEW caught in sorghum and corn fields according to trap type. The Scentry Heliothis traps and Hartstack-wire-white-plastic traps caught 466 and 427 CEW males, respectively, representing the highest percentages, followed by the Universal multicolor bucket trap (275 males). These 3 trap types were not significantly different from each other (Fig. 2A,B, Supplementary Table S2), but their trap catches were significantly higher than 83 and 186 males caught in the Universal unicolor bucket and Hartstack-wire traps, respectively (Fig. 2A,B, Supplementary Table S2).

**Table 1.** Comparison of *H. zea* male catches by trap design between sorghum and corn fields in summer 2022.

Trap	Crop	Mean±SEM % per block	Crop		Position		Crop*Position	
			*F*-value	*P*-value	*F-*value	*P*-value	*F-*value	*P-*value
Unicolor bucket	Sorghum	6.4 ± 1.1	0.00 (1)	0.95	1.11 (4)	0.39	0.29 (4)	0.88
	Corn	7.5 ± 1.6						
Multicolor bucket	Sorghum	21.0 ± 2.1	1.23 (1)	0.29	0.67 (4)	0.62	0.57 (4)	0.69
	Corn	25.6 ± 2.8						
Hartstack-wire	Sorghum	13.6 ± 2.3	0.61 (1)	0.45	0.13 (4)	0.97	1.51 (4)	0.25
	Corn	11.6 ± 2.2						
Hartstack-gray-white plastic	Sorghum	26.8 ± 2.4	0.33 (1)	0.57	0.55 (4)	0.70	1.06 (4)	0.41
	Corn	28.7 ± 2.3						
Scentry Heliothis	Sorghum	32.1 ± 3.0	1.91 (1)	0.19	0.66 (4)	0.64	1.07 (4)	0.41
	Corn	26.5 ± 2.3						

Numbers in parenthesis show the degrees of freedom.

The difference in *H. zea* male catches between sorghum (*n* = 12) and corn (*n = *13) fields and the interaction between crop and trap position were not significant across trap design following *lmm* analysis with crop and trap position as fixed effects, *α*=0.05.

### Trap Color Enhances CEW Catches

**Fig. 3.**
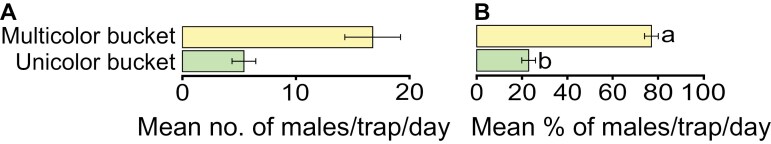
Effect of bucket trap color on trap catch of *Helicoverpa zea* males. The bar plots show (A) the mean number and (B) mean percentage of males caught per trap per day. The traps were baited with sex pheromone and tested in a pairwise design on 25–28 July 2023 (*n* = 12 per trap type). Different letters indicate significant differences following *lmm* and post-hoc analysis with *α=* 0.05. Measures of variation represent ±SEM.

Given the observed significant difference between the unicolor and multicolor bucket traps (Fig. 2A,B, Supplementary Table S2), we carried out a field study to independently validate the observation. We directly compared Universal unicolor *vs.* multicolor bucket traps in a pairwise experiment 25–28 July 2023 (relatively bright moon); all traps were baited with Trécé CEW lures. Catches were significantly different between the trap types (*lmm*, *F* = 74.80, df = 1, *P* < 0.001). A total of 266 CEW males were caught, with the multicolor bucket trap catching more than 3-fold the number of males caught in the unicolor bucket trap (201 vs. 65) (Fig. 3A,B).

We then sought to determine whether the white color of the Scentry Heliothis trap and the white plastic top of the Hartstack trap contributed to their effectiveness (Fig. 2A,B, Supplementary Table S2). We set a similar trapping experiment between 3 and 14 September 2022 (bright moon light), but with some modifications. We discontinued the use of the Universal unicolor bucket trap because it trapped the fewest males, but retained the Universal multicolor bucket trap, Scentry Heliothis trap, and Hartstack-wire trap. In addition, to mimic the all-white Scentry Heliothis trap, we added a modified white Hartstack trap (Hartstack-white-white cheesecloth). There was a significant effect of trap type on the number of CEW males trapped (*lmm*, *F* = 42.28, df = 4, *P* < 0.001). The highest numbers of CEW males were trapped in Hartstack-white-white cheesecloth (741) and Scentry Heliothis (418) traps, both of which also had the highest percentages of all trapped males within each block (Fig. 2C,D). The numbers of males trapped in these 2 trap types were significantly higher than males trapped in the Universal multicolor bucket (146), Hartstack-wire (112), and Hartstack-wire-white cheesecloth (196) traps (Fig. 2C,D, Supplementary Table S3). No significant differences were observed between the Universal multicolor bucket, Hartstack-wire, and Hartstack-wire-white cheesecloth traps (Fig. 2C,D, Supplementary Table S3).

**Fig. 4.**
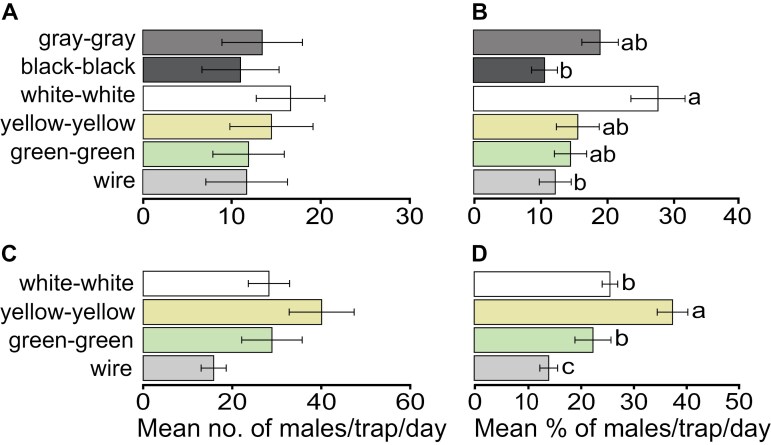
Effect of color on Hartstack trap catch of *Helicoverpa zea* males. The bar plots show (A) the mean number and (B) mean percentage of males caught per trap per day across six Hartstack traps tested in a corn field on 1–6 August 2023 (*n* = 12 per trap type), and (C) the mean number and (D) mean percentage of males caught per trap per day across 4 Hartstack traps tested in a sorghum field on 9–14 August 2023 (*n* = 12 per trap type). All traps were baited with sex pheromone. Except for the wire traps, all other traps were wrapped with dyed cheesecloth (see Fig. 1F). Different letters indicate significant differences following *lmm* and post-hoc analysis with *α=* 0.05. Measures of variation represent ± SEM.

To determine whether color influences Hartstack trap catches, we set out 6 Hartstack traps wrapped in differently colored cheesecloths (Fig. 1H) during 1 to 6 August 2023 (bright moon light). We caught a total of 952 CEW males. There was a significant difference in CEW captured by trap color (*lmm*, *F* = 3.874, df = 5, *P* = 0.004). The highest catch was in Hartstack-white-white (200), the percentage of which considering each block, was significantly higher than in Hartstack-wire and Hartstack-black-black traps (141 and 132 males, respectively), but not different from Hartstack-gray-gray, Hartstack-green-green and Hartstack-yellow-yellow traps (162, 143, and 174 males, respectively) (Fig. 4A,B, Supplementary Table S4).

In a follow-up experiment, we discontinued the use of the gray and black traps to concentrate on colored traps. The corn used for the earlier experiments in 2023 had matured and was ready for harvesting. Therefore, the third experiment was conducted between 9 and 14 August 2023 (low moon light) in a sorghum field. Four Hartstack traps with colored cheesecloths (yellow-yellow, green-green, or white-white) or without cheesecloth (gray wire), were set out in blocks. We trapped 1,357 CEW males, with trap catches significantly influenced by trap color (*glmer* and ANOVA: χ^2^=^45.41, df^ = 3, *P* < 0.001). Following a post-hoc comparison, there were significant differences between traps (Supplementary Table S5). Hartstack-yellow-yellow caught the most males (481), which was significantly higher than all the other traps (Fig. 4C,D, Supplementary Table S5). No significant difference was observed between Hartstack-white-white (339 males) and Hartstack-green-green (347 males), both of which caught significantly more CEW males than Hartstack-wire traps (190 males) (Fig. 4C,D, Supplementary Table S5).

### Correlation Between Trap Capture and Trap Brightness

**Fig. 5.**
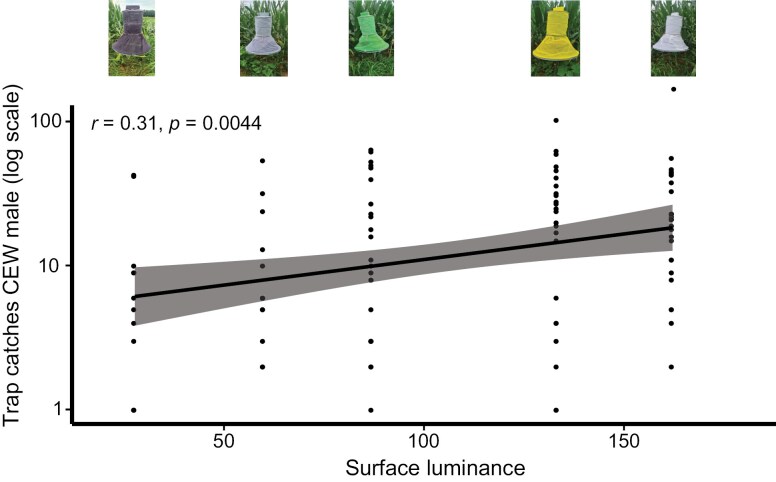
Correlation between trap captures and luminance. The plot shows Pearson’s correlation between trap catches of *H. zea* males presented on a log scale and the luminance (brightness) of the fabrics used to wrap Harststack traps in summer 2023. Each dot represents a trap catch. The black line represents the line of best fit for the model y=10.05+0.09∗luminance. The gray shaded area indicates 95% confidence interval at *α=* 0.05 (*r* = 0.31, *P* = 0.004). The traps, from left to right, are Black, Gray, Green, Yellow, White.

With the colors we perceived as “brighter” trapping more *H. zea* males than “darker” colors, we tested whether there was a relationship between the trap captures and the relative luminance (brightness) of the traps. We observed a significant positive correlation between trap captures and trap brightness (Pearson’s correlation, *r* = 0.31, *P* = 0.004) (Fig. 5), with the best fit line y=10.05+0.09∗luminance. The correlation shows that trap catches for CEW increase with greater grayscale brightness of the trap.

## Discussion

Olfaction predominates sensory repertoires in nocturnal moths, with sex pheromones guiding orientation of males to females over considerable distances (Witzgall et al. 1999, Cardé 2016). For day-active moths and some species of nocturnal moths, visual stimuli are also critical in locating mating partners, plant hosts, and flowers (Doane 1968, Hidaka 1972, Prokopy and Owens 1983, Raguso and Willis 2002, Willis et al. 2011). Therefore, while the advantage of color vision is less obvious in nocturnal moths than in diurnal moths, investigating its significance beyond visual-flow-field phenomena (Kennedy 1940, Cardé 2016) can enhance our understanding of orientation in nocturnal moths. At close range, the presence of a dead or mock female was reported to enhance localization of a pheromone source by male CEW (Carpenter and Sparks 1982), but it is not clear whether males use visual signals or take advantage of local air turbulence generated by the target. Notwithstanding, it is unknown whether visual cues impact pheromone-baited traps for monitoring CEW. We hypothesized that visual contrast between traps and crop background affects the efficiency of pheromone-baited traps for capturing CEW. We found that the capture rate of CEW males varied depending on the trap type and color. Trap colors with brighter shades of gray captured more CEW than trap colors with darker shades of gray.

### Trap Type and Design Influence Male CEW Catches

Bucket traps are commonly used in monitoring noctuid moths (Cardé et al. 2018, Whitfield et al. 2019, Fleming et al. 2021, Saveer et al. 2023). We found that in the presence of different traps, the multicolor bucket trap captured about 2-fold more CEW than the unicolor bucket trap (Fig. 2A,B, Supplementary Fig. S3A). The effectiveness of the multicolor bucket traps became more apparent in a paired design as the multicolor bucket traps caught about 4-fold more CEW males than the unicolor bucket traps (Fig. 3A,B, Supplementary Fig. S4A). Importantly, this pattern was consistent despite the daily population changes and in 2 crops, sorghum and corn (Supplementary Figs. S3A,B and S4A), and under low and bright moon light, indicating that the multicolor bucket trap performs more effectively than the unicolor bucket trap. Since both traps have identical dimensions and design, and were baited with the same lure, it is likely that the yellow-green-white visual spectrum of the multicolor traps enhanced contrast under night conditions as opposed to the visual spectrum of the dark green-colored unicolor traps, making multicolor bucket traps a more apparent visual target for CEW. These results are consistent with a previous observation that 2 other nocturnal noctuid moths—the fall armyworm (*S. frugiperda*) and the velvetbean caterpillar (*A. gemmatalis*)—preferred multicolored over green bucket traps (Mitchell et al. 1989). It remains to be determined whether the contrast of multiple colors plays a role, or whether all-white or all-yellow bucket traps would outperform the multicolor bucket traps.

Since the 1970s, the Hartstack trap has been used as a gold standard for monitoring CEW (Hartstack et al. 1979, Gauthier et al. 1991). Except for a few trapping days (Fig. S3), Hartstack traps caught more *H. zea* than bucket traps when both were in the same experimental block (Fig. 2). The Scentry Heliothis trap, which is also used to monitor heliothine moths, also captured more CEW than bucket traps (Fig. 2). The Hartstack and Scentry Heliothis traps have similar designs but are distinct from bucket traps, which contained strips of insecticide that were not used in the Hartstack and Scentry Heliothis traps. It is possible that the insecticide strip might in some way diminish the effectiveness of the bucket trap (eg repellency), but this is unlikely to account for the daily and season-long patterns we observed. In both the Hartstack and Scentry Heliothis traps, lures are more exposed than in the bucket trap, and the bottom cones of these 2 traps have wide openings. Thus, it is possible that their designs enhance emission and influence the shape of the pheromone plume, promoting the entry of attracted male moths. Trap design had been shown to influence plume pattern, with trap catches of *Cydia nigricana* (Steph) being dependent on the plume pattern (Lewis and Macaulay 1976). The Scentry Heliothis trap was more effective than the Hartstack-wire trap, in contrast to an earlier observation in which Hartstack trap, similar to the Hartstack-wire in our study, captured more CEW than the Scentry Heliothis trap (Gauthier et al. 1991). We used a similar lure type, Pherocon (Trécé) lure, as Gauthier et al. (1991), but with a different field trapping design, which could explain the observed differences. For instance, to maximize trap catches, an optimal trap positioning is required (Prokopy and Owens 1983). Therefore, differing trap positioning could have caused variation between the 2 studies. Also of note is that the pliable and collapsible nylon Scentry Heliothis trap is more susceptible to accidental constriction or even blockage of the entry hole to the upper portion of the trap.

### Bright Colors Enhance Male CEW Catches

The inclusion of a white plastic top significantly increased the capture rate of Hartstack traps, with a similar effectiveness relative to the Scentry Heliothis trap (Fig. 2B). Intriguingly, when Hartstack-wire traps were wrapped in white cheesecloth, they captured about twice as many moths as the Scentry Heliothis traps and 5-fold more than the Hartstack-wire traps (Fig. 2D). Considering that the Scentry Heliothis trap is made of white nylon net, the significant increase in trap catches with Hartstack-white-white cheesecloth traps provide further evidence that CEW males exploit visual contrast to locate a pheromone source. Because white color enhanced trap catches, we improvised Hartstack-wire traps and investigated whether gray, black, green and yellow affect catches of CEW. On brightly lit (full moon) nights, the highest percentage of CEW males were trapped in Hartstack-white-white traps, and the fewest CEW were trapped in Hartstack-black-black traps (Fig. 4B). In a follow-up trapping assay, under low moonlight, with only gray wire, green, yellow and white Hartstack traps, the highest percentage of CEW were caught in Hartstack-yellow-yellow traps, followed by Hartstack-white-white traps (Fig. 4D). Since we showed that neither crop nor the interaction of crop and position affects trap catches (Table 1), we suspect that both traps appear visually similar to CEW males, while bright moonlight might reduce the overall trap catch (compare Fig. 4A (bright moonlight) and Fig. 4C (dim moonlight)). Moreover, our comparison of colors in cheesecloth-wrapped traps eliminated the potential effects of the cheesecloth on pheromone emission from the traps and the shape of the emitted pheromone plume. Thus, cheesecloth (trap) color significantly affected trap catch, independently of trap design.

The brightness (luminance) of the cheesecloths used to wrap Hartstack traps ranged from 27.6 to 166.6 (Supplementary Table S1), with black and white as the darkest and brightest fabrics, respectively. Brightly colored pheromone-baited traps likely have greater contrast with the background foliage, particularly at night. We postulate that CEW has an innate ability to discriminate between shades of gray. Our hypothesis is supported by the positive correlation between trap captures and luminance. Although we did not determine the luminance of the bucket traps, it is likely that the yellow funnel lid and the white bucket segment give multicolored bucket traps characteristically brighter shades of gray in minimal night-time illumination. Consequently, more CEW are attracted and trapped, which further supports the luminance hypothesis.

### Implication of Visual Contrast in CEW Communication

*Helicoverpa zea* in our study, as well as *S. frugiperda* and *A. gemmatalis* (Mitchell et al. 1989), are attracted to traps with high spectral reflectance. In contrast, the cabbage looper (*Trichoplusia ni* Hübner), soybean looper (*Pseudoplusia includens* Walker), and codling moth (*Cydia pomonella* Linnaeus) are attracted to traps with low reflectance (McLaughlin et al. 1975, Coudriet and Henneberry 1976, Knight and Miliczky 2003). These observations underscore interactions and potential synergism between visual and olfactory stimuli that could be widespread beyond the well-studied hawk moth species (Raguso and Willis 2002, Warrant and Somanathan 2022). In the context of sexual communication in moths, which has been exploited in mating disruption to manage pest populations, little is known about the role of vision except for the visual feedback system used by moths in navigation (Kennedy and Marsh 1974, Vickers and Baker 1994, Cardé 2016). In day-light conditions, many insect species have chromatic (color) vision; however, under night-time low illumination, vision is largely achromatic and limited to shades of gray and white. If the ability to discriminate between shades of gray is innate and if nocturnally active *H. zea* males incorporate visual contrast to locate pheromone sources, we would expect that brighter shades of gray and white objects would synergize courtship and mating, a fundamental knowledge gap that requires elucidating. Notably, the wings of *H. zea* females are light yellow-brown colored and would appear light gray at night. Perhaps males evolved a mate-finding system that integrates olfactory orientation to the female sex pheromone with visual signals associated with the female’s wings. It is also possible that visual discrimination of colors and luminance evolved in the context of flower and nectar foraging by both sexes. In this context, male moths of a closely related allopatric species, the cotton bollworm (*Helicoverpa armigera* Hübner) (Mitter et al. 1993, Behere et al. 2007), have an innate preference for blue color in an ambient illumination of approximately 2 lux (full moon illumination is approximately 1 lux), but this preference is overridden upon learning yellow color (Satoh et al. 2016). This poses several questions: Do *H. zea* discriminate colors under full-moon conditions? Do males express different color preferences when foraging for nectar and in the presence of female sex pheromone? If the visual preferences of males can be shifted through learning in a foraging context (as in *H. armigera*), can visual preference be altered in a mate-finding context, or is visual preference fixed in sexual orientation? While addressing these questions, future studies should take into consideration the proximity of traps to foliage, trap apparency and position relative to the canopy, all of which can significantly impact trap catches (Lewis and Macaulay 1976, Prokopy and Owens 1983).

Whether used in surveillance (early detection), monitoring (population estimation) or active pest suppression (mass trapping, attract-and-kill, mating disruption), more effective traps that capture more adult moths are preferred to less effective traps. Our study underscores the importance of visual contrast in optimizing traps of nocturnal pests. Similar to the management of diurnal pests where visual stimuli are incorporated into attractive and disruptive traps (Prokopy and Owens 1983), such information can be exploited to improve existing technologies as well as develop new trapping devices targeting nocturnal agricultural and forest pests.

## Supplementary material

Supplementary material is available at *Journal of Economic Entomology* online.

toaf024_suppl_Supplementary_Material

## Data Availability

All relevant data can be found within the article, its supplementary material and in the provided dataset.
