# Supplementary material for: Visual cues enhance effectiveness of pheromone-baited traps for the corn earworm moth, Helicoverpa zea (Lepidoptera: Noctuidae)
Source: J Econ Entomol. 2025 Feb 13;118(2):662–71. doi: 10.1093/jee/toaf024 (PMC12034308; doi:10.1093/jee/toaf024)
Supplement: toaf024_suppl_Supplementary_Material [file toaf024_suppl_supplementary_material.docx]

Supplementary material

**Visual cues enhance effectiveness of pheromone-baited traps for the corn earworm moth, *Helicoverpa zea* (Lepidoptera: Noctuidae)**

**Charles A. Kwadha^1,5,*^, Ahmed M. Saveer^1,2,*^, Ayako Wada-Katsumata^1^, Dominic D. Reisig^1^, Gabriel P. Hughes^3,4^, Ring T. Cardé^3^, Coby Schal^1,5^**

^1^Department of Entomology and Plant Pathology, North Carolina State University, Raleigh, NC 27695, USA, ^2^Present address: Invasive Insect Biocontrol and Behavior Laboratory, United States Department of Agriculture, Agricultural Research Service, Beltsville, MD 20705, USA, ^3^Department of Entomology, University of California Riverside, Riverside, CA 92521, USA, ^4^Present address: Animal and Plant Inspection Service, United States Department of Agriculture, Agricultural Research Service, Riverdale, MD 20737, USA, *The authors contributed equally, and ^5^Corresponding authors, emails: [ckwadha@ncsu.edu](mailto:ckwadha@ncsu.edu), [coby@ncsu.edu](mailto:coby@ncsu.edu)

**Table S1.** Red, green, and blue (*rgb*) mean values for cheesecloth fabrics as determined by ImageJ. See Material and Methods for detailed description (related to Figs. 1,5).

| Fabric color | Area | Mean | Minimum | Maximum | % |
| --- | --- | --- | --- | --- | --- |
| Black | 419904 | 27.7 | 0 | 72 | 99.79 |
| Gray | 419904 | 59.9 | 0 | 110 | 99.99 |
| Green | 419904 | 87.0 | 0 | 181 | 100.00 |
| Yellow | 419904 | 133.3 | 0 | 255 | 99.92 |
| White | 419904 | 162.1 | 0 | 255 | 99.98 |

For each fabric color, an equal area (in square pixels) was selected. From the area, we obtained average gray values of all selected pixels. The lowest and highest gray values within the selected areas are represented by minimum and maximum values, respectively. The percentage (%) represents the proportion of non-zero pixels in the selected area.

**Table S2.** Multiple comparisons of male CEW trap catches in 2022 (related to Fig. 2A,B).

| Trap | Z-value | *P-*value |
| --- | --- | --- |
| Scentry *vs.* Hartstack-wire-white plastic | 0.553 | 1.000 |
| Scentry *vs.* Multicolor | 0.336 | 1.000 |
| Scentry *vs.* Hartstack-wire | 4.916 | 8.83e-06*** |
| Scentry *vs.* Unicolor | 6.667 | 2.61e-10*** |
| Hartstack-wire-white plastic *vs.* Multicolor | 0.889 | 1.000 |
| Hartstack-wire-white plastic *vs.* Hartstack-wire | 5.466 | 4.61e-07*** |
| Hartstack-wire-white plastic *vs.* Unicolor | 7.214 | 5.45e-12*** |
| Multicolor *vs.* Hartstack-wire | 4.577 | 4.71e-05*** |
| Multicolor *vs.* Unicolor | 6.327 | 2.50e-09*** |
| Hartstack-wire *vs.* Unicolor | 1.748 | 0.805 |

Tukey’s test with Bonferroni adjustment was used as a post-hoc test to establish significant differences.

Hartstack-wire refers to the standard gray wire Hartstack trap. Hartstack-wire-white plastic refers to the standard gray wire Hartstack trap with a white plastic trap on top.

**Table S3.** Multiple comparisons of male CEW trap catches in 2022 (related to Fig. 2C,D).

| Trap | Z-value | *P-*value |
| --- | --- | --- |
| Hartstack-white-white cheesecloth *vs.* Scentry Heliothis | 5.348 | 8.88e-07*** |
| Hartstack-white-white cheesecloth *vs.* Hartstack-wire-white cheesecloth | 8.604 | < 2e-16*** |
| Hartstack-white-white cheesecloth *vs.* Multicolor bucket | 10.090 | < 2e-16*** |
| Hartstack-white- white cheesecloth *vs.* Hartstack-wire | 11.561 | < 2e-16*** |
| Scentry Heliothis *vs.* Hartstack-wire-white cheesecloth | 3.256 | 0.0113* |
| Scentry Heliothis *vs.* Multicolor bucket | 4.741 | 2.12e-05*** |
| Scentry Heliothis *vs.* Hartstack-wire | 6.213 | 5.21e-09*** |
| Hartstack-wire-white cheesecloth *vs.* Multicolor bucket | 1.485 | 1.0000 |
| Hartstack-wire-white cheesecloth *vs.* Hartstack-wire | 2.957 | 0.0311* |
| Multicolor bucket *vs.* Hartstack-wire | 1.471 | 1.0000 |

Tukey’s test with Bonferroni adjustment was used as a post-hoc test to establish significant differences.

Hartstack-wire refers to the standard gray wire Hartstack trap. Hartstack-wire-white cheesecloth refers to the standard gray wire Hartstack trap with the top wire section wrapped in white cheesecloth.

**Table S4.** Multiple comparisons of male CEW trap catches between Hartstack traps used in a corn field 1–6 August 2023 (related to Fig. 4A,B).

| Trap (Hartstack) | Z-value | *P-*value |
| --- | --- | --- |
| white-white *vs.* gray-gray | 1.617 | 1.000 |
| white-white *vs.* yellow-yellow | 2.775 | 0.083 |
| white-white *vs.* green-green | 2.775 | 0.083 |
| white-white *vs.* wire | 3.476 | 0.007** |
| white-white *vs.* black-black | 3.769 | 0.002** |
| gray-gray *vs.* yellow-yellow | 1.158 | 1.000 |
| gray-gray *vs.* green-green | 1.158 | 1.000 |
| gray-gray *vs.* wire | 1.859 | 0.946 |
| gray-gray *vs.* black-black | 2.151 | 0.472 |
| yellow-yellow *vs.* green-green | 0.000 | 1.000 |
| yellow-yellow *vs.* wire | 0.701 | 1.000 |
| yellow-yellow *vs.* black-black | 0.993 | 1.000 |
| green-green *vs.* wire | 0.701 | 1.000 |
| green-green *vs.* black-black | 0.994 | 1.000 |
| wire *vs.* black-black | 0.292 | 1.000 |

Tukey’s test with Bonferroni adjustment was used as a post-hoc test to establish significant differences.

The first color refers to the color of the cheesecloth wrapped around the bottom cone of the Hartstack-wire trap. The second color refers to the color of the cheesecloth wrapped around the top container of the Hartstack-wire trap.

**Table S5.** Multiple comparisons of CEW trap catches between Hartstack traps used in a sorghum field on 9–14 August 2023 (related to Fig. 4C,D).

| Trap (Hartstack) | Z-value | *P-*value |
| --- | --- | --- |
| yellow-yellow *vs.* white-white | 2.615 | 0.044* |
| yellow-yellow *vs.* green-green | 0.3780 | 0.001** |
| yellow-yellow *vs.* wire | 0.6631 | <0.001*** |
| white-white *vs.* green-green | 1.164 | 0.6497 |
| white-white *vs.* wire | 4.086 | 0.000*** |
| green-green *vs.* wire | 2.925 | 0.018* |

Tukey’s test with Bonferroni adjustment was used as a post-hoc test to establish significant differences.

The first color refers to the color of the cheesecloth wrapped around the bottom cone of the Hartstack-wire trap. The second color refers to the color of the cheesecloth wrapped around the top container of the Hartstack-wire trap.

**
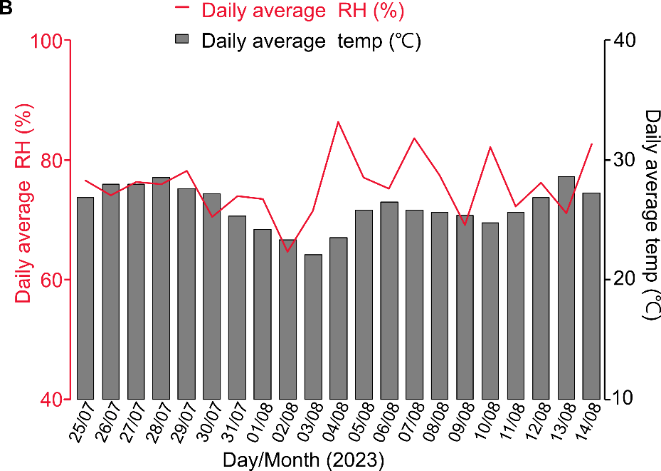

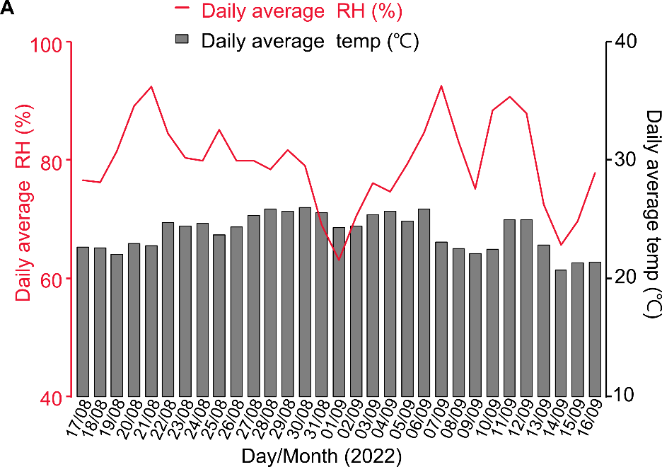
**

**Fig. S1.** Daily average temperature (℃) and relative humidity (RH, %) for the field trapping period in 2022 and 2023. (A) 17 August to 16 September 2022. (B) 25 July to 14 August 2023. The weather data were recorded by the North Carolina State Climate Office (<https://products.climate.ncsu.edu/cardinal/request>, accessed October 10, 2023).The red lines and gray bars indicate the daily average %RH and average temperature (℃), respectively.

**Fig. S2.** Scanned sections of cheesecloth fabrics. Fabrics were scanned at 600 dots per inch (*dpi*) with (A) autocolor mode and (B) grayscale mode. A section (2.5 x 1.5 cm) of each of the fabrics was used to establish the *red, green* and *blue* mean gray values for each color using ImageJ.


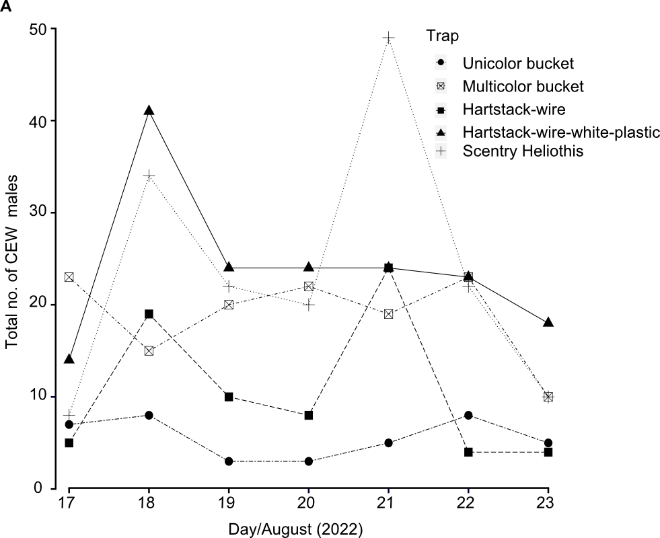


**
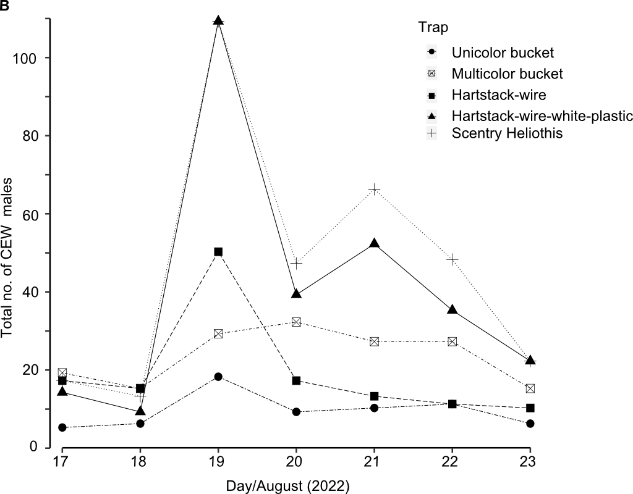
**

**
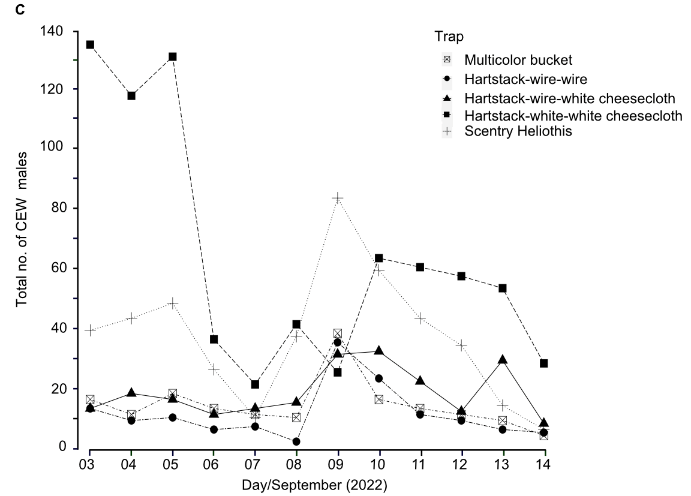
**

**Fig. S3.** The daily pattern of total catches of *Helicoverpa zea* per trap design in summer 2022. Traps were set in (A) corn and (B) sorghum fields between 17 and 23 August, and in a sorghum field (C) between 3 and 14 September. Three trap designs were used: Universal bucket trap (unicolor and multicolor), Hartstack trap (Hartstack-wire and Hartstack-wire-white plastic), and Scentry Heliothis trap. *n* = 2–4 and 1–2 traps per design/day for the first and second trapping periods, respectively.

**
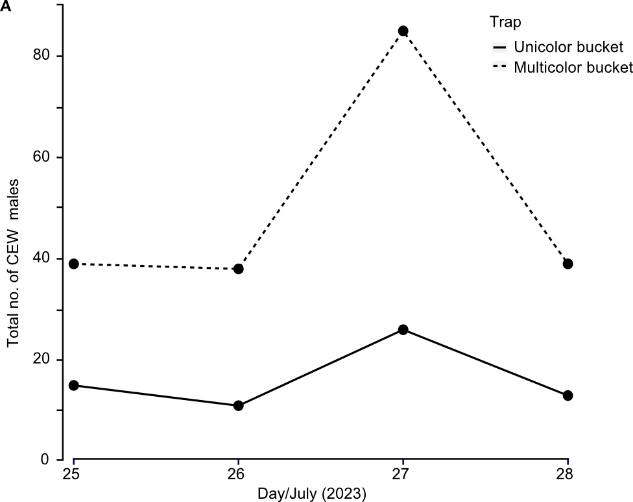
**

**
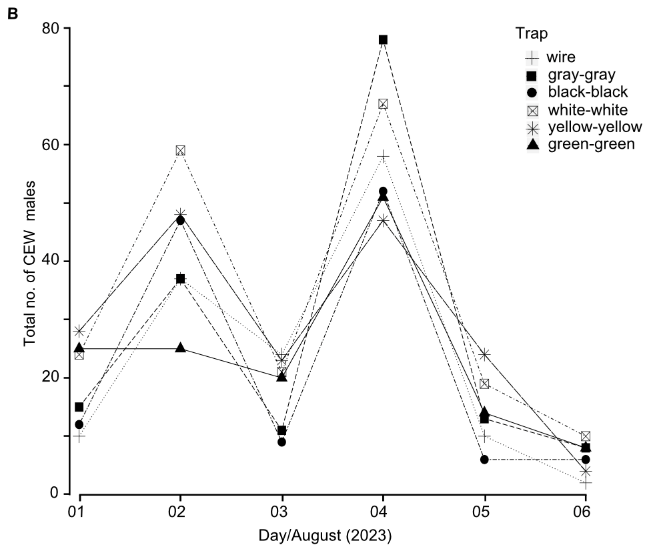

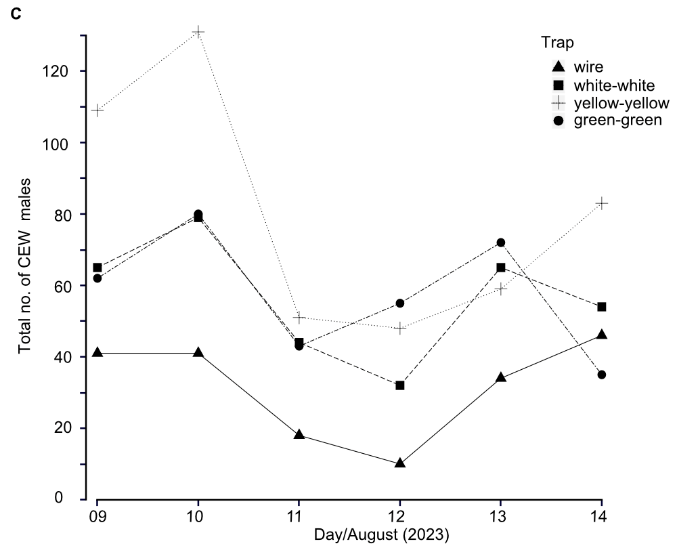
**

**Fig. S4.** The daily pattern of total catches of *Helicoverpa zea* per trap design in summer 2023. Traps were set in corn fields (A) 25–28 July, (B) 1–6 August, and (C) in a sorghum field 9–14 August. In the first trapping period (A), Universal bucket traps (unicolor and multicolor) were used in a paired design. In the second period (B) six Hartstack traps with modified color were used: Hartstack-wire; -gray-gray; -black-black; -white-white; -yellow-yellow; -green-green. In the third period (C) four Hartstack traps with modified color were used: Hartstack-wire; -white-white; -yellow-yellow; -green-green. Except for the wire traps, all other traps in (B) and (C) were wrapped with dyed cheesecloth. *n* = 2–3 traps per type/day.
